# CP-BayesNE: Uncertainty-Aware Personalized Nutrient-Density Estimation from Food Images Using Conformal Prediction and Prior-Corrected Dietary Adaptation

**DOI:** 10.3390/nu18213567

**Published:** 2026-10-28

**Authors:** Zhaozhen Tong, Kumiko Ono, Shu-Chun Lee, Hsin-Yen Yen, Masahide Nakamura, Sinan Chen

**Affiliations:** 1 Graduate School of Engineering, Kobe University, 1-1 Rokkodai-cho, Nada-ku, Kobe 657-8501, Japan; 243t804t@gsuite.kobe-u.ac.jp (Z.T.); masa-n@cmds.kobe-u.ac.jp (M.N.); 2 Graduate School of Medicine, Kobe University, 7-10-2 Tomogaoka, Suma-ku, Kobe 654-0142, Japan; onoku@tiger.kobe-u.ac.jp; 3 School of Gerontology and Long-Term Care, Taipei Medical University, No. 250, Wuxing St., Taipei 11031, Taiwan; sclee@tmu.edu.tw (S.-C.L.); kenji@tmu.edu.tw (H.-Y.Y.); 4 Center for Mathematical and Data Sciences, Kobe University, 1-1 Rokkodai-cho, Nada-ku, Kobe 657-8501, Japan

**Keywords:** dietary assessment, food image recognition, precision nutrition, AI-driven personalized nutrition, conformal prediction, Bayesian adaptation, nutrient estimation, uncertainty calibration

## Abstract

Background/Objectives: Reliable longitudinal dietary assessment is a prerequisite for precision nutrition, important for studying nutrition-related changes in health and physical function, but conventional dietary recording can impose substantial user burden. AI-driven, food-image-based assessment can reduce this burden, although uncertainty in food recognition and inter-individual dietary differences may propagate to nutrient estimation. Methods:We developed CP-BayesNE, an uncertainty-aware framework that combines temperature-scaled food classification, conformal candidate sets, training-prior-corrected dietary-history personalization, and posterior-weighted nutrient estimation to support AI-driven personalized nutrition. Results: EfficientNetV2-S achieved 78.01% Top-1 and 93.67% Top-5 accuracy, while temperature scaling reduced expected calibration error from 0.11238 to 0.04345. At α=0.10, regularized adaptive prediction sets reduced the average candidate-set size from 80.78 to 5.77 while maintaining 94.32% empirical category coverage. In FoodLogAthl-218, prior-corrected personalization reduced nutrient density mean absolute error (MAE) by 4.0–6.6% across energy and macronutrients over 51 users. A single-participant 92-meal personal dataset provided preliminary end-to-end feasibility evidence, while also revealing limitations, including slightly worse fat MAE and conservative nutrient intervals. Conclusions: These findings support uncertainty-aware personalized dietary assessment while emphasizing that category-level conformal validity does not directly imply accurate true dietary intake.

## 1. Introduction

Reliable dietary assessment is a foundation of precision nutrition [1,2] and is essential for understanding nutritional behavior and its relationships with health outcomes over time. In particular, longitudinal dietary information can support the study of energy and macronutrient patterns in free-living settings and may ultimately be integrated with complementary measures of physical function, gait, body composition, and skeletal-muscle status in multimodal health-monitoring frameworks [3,4,5]. Self-reported approaches, including food diaries and food records, dietary recall methods such as the 24 h recall, and food-frequency questionnaires, are widely used for dietary assessment and have an established methodological basis [6]. In repeated, longitudinal, or free-living use, however, these methods can be affected by participant burden, reliance on memory, reporting errors, omitted foods, and portion-size estimation errors, particularly when sustained recording is required over time [7,8]. Image-assisted and image-based dietary assessment has therefore been investigated as a way to reduce reliance on active recall and to lower the burden of repeated recording [7], and recent artificial-intelligence-based methods increasingly seek to extract dietary information directly from food images [9,10,11].

Image acquisition alone, however, does not guarantee reliable dietary inference. Automated food recognition from images remains imperfect, and a classifier may assign meaningful probability to more than one plausible category rather than a single unambiguous one; for example, when dishes are visually similar [12,13]. A conventional Top-1 decision collapses this uncertainty into a single category, discarding the information carried by the remaining plausible categories. Probability calibration can improve the interpretability of the resulting predictive probabilities [14], and conformal prediction then provides a model-agnostic way to construct prediction sets with a target marginal coverage under appropriate exchangeability assumptions [15,16,17], retaining a statistically controlled set of plausible food categories rather than committing prematurely to a single class. This coverage guarantee presupposes that the calibration and deployment conditions are exchangeable, and distributional shifts between the two can weaken it in practice.

Even when a conformal candidate set retains several plausible categories, different users have different dietary habits, and a user’s previous food records can therefore provide informative prior information when multiple visually plausible categories remain. Previous work has explored personalized food-image classification [18], and real-world dietary datasets such as FoodLogAthl-218 enable user-level evaluation of personalized recognition strategies [19]. A key methodological issue must, however, be addressed explicitly: the discriminative classifier posterior already reflects the class prior of the training distribution, so directly multiplying this posterior by a personal frequency prior can conceptually double-count prior information already embedded in the classifier output. A more defensible adaptation first removes the training-distribution prior and then introduces the user-specific prior under a prior-shift interpretation [20,21,22]; this prior-corrected replacement is precisely the personalization mechanism used in the present framework. A further challenge then appears downstream of category recognition: even when the true food class is included in a conformal candidate set, this does not directly guarantee coverage or accuracy for the derived nutrient values, which additionally depend on food-composition references, candidate weights, portion assumptions, and aggregation across multiple meal items [23,24]. Category-level and nutrient-value uncertainty must therefore be treated as distinct inferential layers, calibrated separately rather than assumed to coincide.

This study develops and evaluates CP-BayesNE (Conformal Prediction–Bayesian personalization–Nutrient Estimation), a framework that integrates calibrated food recognition and conformal candidate sets to address category-level uncertainty, prior-corrected dietary-history personalization to incorporate individual habits without double-counting the training prior, and posterior-weighted nutrient estimation together with an independently calibrated nutrient-value interval to address the downstream uncertainty layer described above. The study uses three complementary data sources: UEC FOOD 256 (UEC-256) for model development and category-level uncertainty analysis; FoodLogAthl-218 for population-level validation across 51 users; and a 92-meal personal dataset for end-to-end evaluation in a real multi-item meal pipeline. These three sources were selected to be complementary rather than redundant: UEC-256 supports controlled classifier and conformal development, FoodLogAthl-218 supports population-level evaluation of the personalization mechanism across many independent users, and the personal meal dataset supports end-to-end validation of the full multi-item pipeline under real meal-recording conditions (Section 3.2). The main objectives are to determine whether: (1) conformal prediction can represent food-category uncertainty with practical candidate-set efficiency (Section 4.1); (2) prior-corrected dietary personalization improves nutrient-density estimation at the user level (Section 4.2); (3) the benefit of personalization depends on the amount of dietary history available (Section 4.3); and (4) downstream nutrient uncertainty requires calibration separate from category-level conformal prediction (Section 4.4). The remainder of the paper is organized as follows: Section 2 reviews related work and positions the present study, Section 3 describes the proposed framework and experimental design, Section 4 reports the results, Section 5 discusses implications and limitations, and Section 6 concludes the paper.

## 2. Literature and Background Works Related to Systematic Dietary Assessments

This section reviews prior work on digital dietary assessment, food-image-based nutrient estimation, personalization, and uncertainty-aware inference. The aim is not to provide a comprehensive survey but to position CP-BayesNE relative to recent studies that are closest to the present objective: low-burden, uncertainty-aware, and personalized nutrient-density estimation from food images.

### 2.1. Digital Dietary Assessment and Precision Nutrition

Accurate dietary assessment remains a central requirement for precision nutrition, nutritional epidemiology, and longitudinal health monitoring. Recent reviews have emphasized that conventional self-report instruments are useful and widely established but can be affected by recall burden, reporting bias, and portion-size misestimation, especially when repeated records are needed in free-living settings [25,26,27]. Digital and technology-enabled approaches have therefore been explored as a way to reduce burden and increase the temporal resolution of dietary data collection [28,29].

Within this broader field, image-assisted and image-based dietary assessment has become one of the most active directions. Scoping and systematic reviews show that food images can support food identification, portion-size estimation, and nutrient estimation but also highlight that performance remains highly dependent on image quality, food complexity, dataset coverage, cultural food diversity, and the availability of reliable reference labels [30,31,32,33]. These observations are directly relevant to the present study because our target is not a one-time controlled meal analysis but a framework that could support repeated dietary monitoring with explicit uncertainty representation. Taken together, these reviews support the premise that reducing dietary-assessment burden through image-based methods is both necessary and still incompletely solved, particularly with respect to representing and propagating the uncertainty inherent in automated food recognition rather than treating it as a solved sub-problem.

### 2.2. Food-Image Recognition and Nutrient Estimation

Most food-image-based dietary assessment pipelines include several steps: food detection or segmentation, food recognition, portion or volume estimation, and nutrient calculation using a food-composition reference. Surveys of image-based food recognition and volume estimation have shown that food classification has improved substantially with deep learning, but portion estimation and nutrient quantification remain difficult, particularly for mixed dishes and foods with hidden ingredients such as oils or sauces [34,35].

Recent studies have attempted to estimate nutritional composition more directly from images. Some works use segmentation and regression to predict calories and macronutrients from food images [36], while others incorporate transformer-based visual backbones for food nutrient recognition [37]. RGB-D and depth-refined systems use additional depth or height information to improve volume- or intake-related estimation [38,39]. Camera-based systems have also been evaluated in institutional or clinical-like settings, where the served portion or menu information can be controlled more easily than in ordinary free-living meals [40].

These studies are important because they show that food-image analysis can support automated dietary assessment. However, many of them focus on improving visual prediction accuracy, segmentation quality, or portion estimation. In contrast, the present study assumes that food recognition remains uncertain and focuses on how such uncertainty should be propagated into nutrient-density estimation. CP-BayesNE therefore does not propose a new image-recognition backbone. Instead, it treats the classifier output as an uncertain input to a downstream dietary-inference layer. Taken together, these studies support the premise that advances in raw recognition accuracy alone are insufficient and motivate the need for an explicit uncertainty-propagation layer connecting food-image recognition to nutrient-density estimation.

### 2.3. Foundation Models and Large Language Models for Dietary Assessment

Recent multimodal large language models and vision–language models have opened a new direction for dietary assessment. Studies using multimodal ChatGPT (GPT-4V or ChatGPT-4) or related models suggest that general-purpose foundation models can recognize food items, infer contextual information, and estimate nutritional content from meal photographs [41,42]. Other work has examined whether artificial-intelligence systems can provide consistent nutritional information compared with nutrition professionals or structured reference [43].

These studies are promising, but they also reinforce a central limitation of current automated dietary assessment: nutrient estimates from food images are sensitive to portion interpretation, prompt design, dataset domain, and reference construction. This is particularly important for free-living images, where true consumed weight is often unavailable. The present study is complementary to foundation-model-based dietary assessment. Rather than relying on an open-ended generative model response, CP-BayesNE uses a fixed food taxonomy, calibrated predictive probabilities, conformal candidate sets, and explicit nutrient-reference mappings. This makes the uncertainty layer more transparent and easier to evaluate at the category and nutrient-density levels. These observations motivate the hypothesis that a structured, probability-calibrated pipeline can offer more evaluable uncertainty guarantees than an open-ended foundation-model response, at the cost of relying on a fixed food taxonomy.

### 2.4. Personalization and Uncertainty in Dietary Inference

Personalization is an important but still underdeveloped component of food-image-based dietary assessment. A user’s dietary history may contain useful information when several visually plausible food categories remain possible. However, naive personalization can be problematic if a discriminative classifier posterior is directly multiplied by a personal frequency prior, because the classifier posterior already reflects the training-distribution class prior. Label-shift and prior-correction methods provide a principled basis for separating likelihood-like evidence from prior information [20,21,22].

Uncertainty quantification is another important requirement for practical dietary assessment. Conformal prediction has been increasingly discussed as a way to attach finite-sample uncertainty guarantees to machine-learning predictions, including in health and clinical contexts [17,44]. For image classifiers, adaptive and regularized conformal prediction sets can represent multiple plausible labels rather than forcing a single Top-1 decision [15,16]. However, a category-level conformal guarantee does not automatically imply reliable nutrient-value intervals, because nutrient estimates also depend on food-composition mappings, candidate weights, portion assumptions, and aggregation across meal items.

The present study combines these two directions. It uses conformal prediction to retain statistically controlled food-category candidate sets, applies prior-corrected dietary-history personalization within those sets, and calibrates nutrient-value uncertainty separately from category-level uncertainty. This design directly addresses the gap between food recognition and downstream nutrient inference. These observations motivate the hypothesis that treating category-level and nutrient-value uncertainty as distinct, separately calibrated inferential layers is necessary, rather than assuming that a valid category-level guarantee automatically extends to the nutrient values derived from it.

### 2.5. Positioning of the Present Study

Table 1 summarizes how CP-BayesNE differs from representative related studies. Existing work has made important progress in digital dietary assessment, food-image recognition, nutrient prediction, foundation-model-based meal analysis, and clinical applications of uncertainty quantification. The distinctive feature of the present study is the combination of four elements in one dietary-inference framework: calibrated food recognition, conformal candidate sets, training-prior-corrected personalization, and separately calibrated nutrient-value uncertainty.

## 3. Materials and Methods

### 3.1. Study Design and Overall Framework

CP-BayesNE was designed as a downstream dietary-inference framework for food images, built on top of an existing classification backbone rather than proposing a new recognition architecture; the contribution lies in the composition of calibration, conformal candidate-set construction, prior-corrected personalization, and independently calibrated nutrient-value uncertainty described below.

For an image crop *x*, the base classifier produces a calibrated posterior probability over the UEC FOOD 256 class space. Conformal prediction constructs a candidate set $C_{\alpha }\left(x\right)$ at miscoverage level α. A user-specific dietary prior is then incorporated after correcting for the class prior represented in the training distribution. Nutrient values are estimated as posterior-weighted averages over the candidate set, and nutrient-value uncertainty is calibrated separately from the category-level conformal set.

The analytical scope of the present study is intentionally narrower than direct measurement of true dietary intake. FoodLogAthl-218 does not contain measured portion or weight labels, and the personal meal dataset uses dish- or product-level declared nutrient values rather than independent weighed-intake measurements. Therefore, the main population-level endpoint is nutrient-density estimation under mapped food references, whereas real-meal results are interpreted as end-to-end numerical errors under the available declared references.

**Figure 1:**
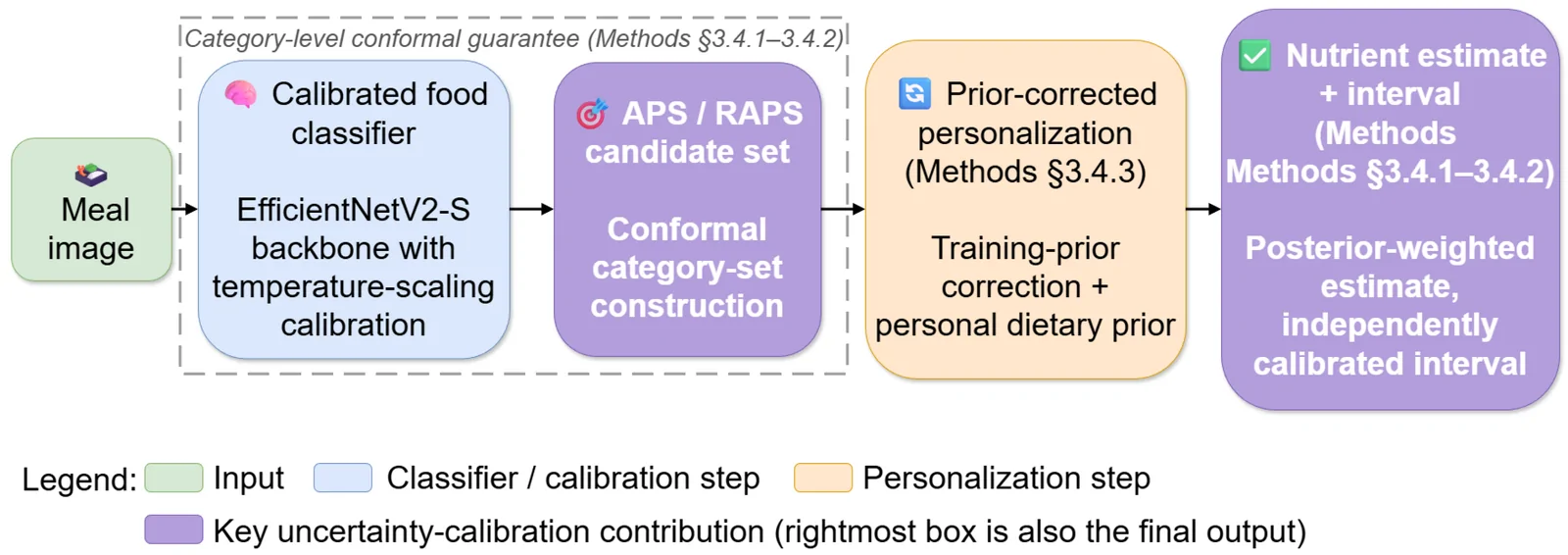
Overall analytical framework of CP-BayesNE. The present study focuses on the dietary-assessment pipeline; future integration with physical-function outcomes is outside the scope of the current evaluation.

Three complementary data sources support this design: UEC FOOD 256 for classifier development and category-level conformal calibration, FoodLogAthl-218 for population-level evaluation of the personalization mechanism across 51 independent users, and the personal meal dataset for end-to-end validation on real multi-item meals. Section 3.2 details the scale, portion-annotation status, and analytical role of each dataset. Figure 1 summarizes the overall pipeline described in the remainder of this section.

### 3.2. Data Sources and Reference Construction

#### 3.2.1. UEC FOOD 256

UEC FOOD 256 (UEC-256) [45] was used for model development, probability calibration, and category-level conformal evaluation. The task contains 256 food categories. The frozen dataset split contained separate training, validation, and test partitions, with all 256 categories represented. The validation logits used for calibration contained 4592 samples. UEC-256 itself does not provide nutrient information; therefore, category labels were linked to a nutrient-reference table derived from the Standard Tables of Food Composition in Japan 2020 (8th Revised Edition) [46]. Per-100 g nutrient values were used as the density reference.

#### 3.2.2. FoodLogAthl-218

FoodLogAthl-218 is a real-world food-image dataset collected using dietary-management applications [19]. Of its 218 original categories, 100 could be mapped to the UEC-256 taxonomy; 118 categories were excluded from the mapped evaluation. Users with at least 30 eligible mapped images were retained, resulting in 51 users. For each user, records were chronologically sorted and split into the first 70% for prior construction and the remaining 30% for evaluation. FoodLogAthl-218 does not contain measured portion or weight labels. Consequently, the reference target was the per-100 g nutrient-density vector associated with the mapped UEC-256 class. This dataset therefore evaluates population-level personalization mechanisms rather than true absolute intake.

#### 3.2.3. Personal Meal Dataset

A personal dataset containing 92 meal events and 402 food-item records was used for end-to-end evaluation in a real multi-item meal pipeline. Of the 92 meal images, 87 were captured using a compact digital camera (model M06; image resolution: 8160 × 6120 pixels; manufacturer information unavailable), and two were captured using an Apple iPhone 12 mini (Apple Inc., Cupertino, CA, USA). The remaining three images lacked identifiable device metadata. The main evaluation includes one individual. Food-item references were finalized through interactive manual review of crop-to-food mappings. Meal-level nutrient references were derived from dish- or product-level declared nutrient values recorded with the meal data. These references do not constitute independently weighed consumed amounts. This dataset therefore provides deeper validation of the operational pipeline and the magnitude of meal-level numerical errors but does not establish population-level absolute intake accuracy.

#### 3.2.4. Dataset Overview and Comparison

**Figure 2:**
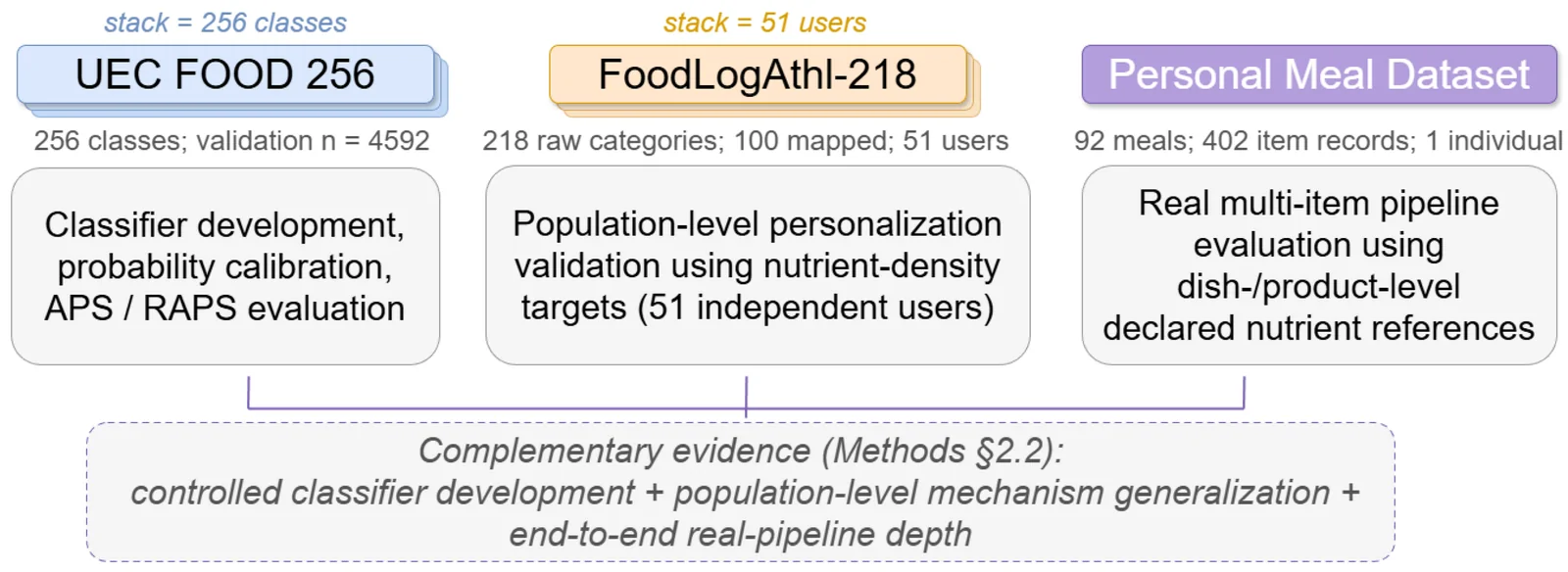
Complementary roles of the three datasets used in the study. Blue, orange, and purple identify UEC FOOD 256, FoodLogAthl-218, and the personal meal dataset, respectively.

**Figure 3:**
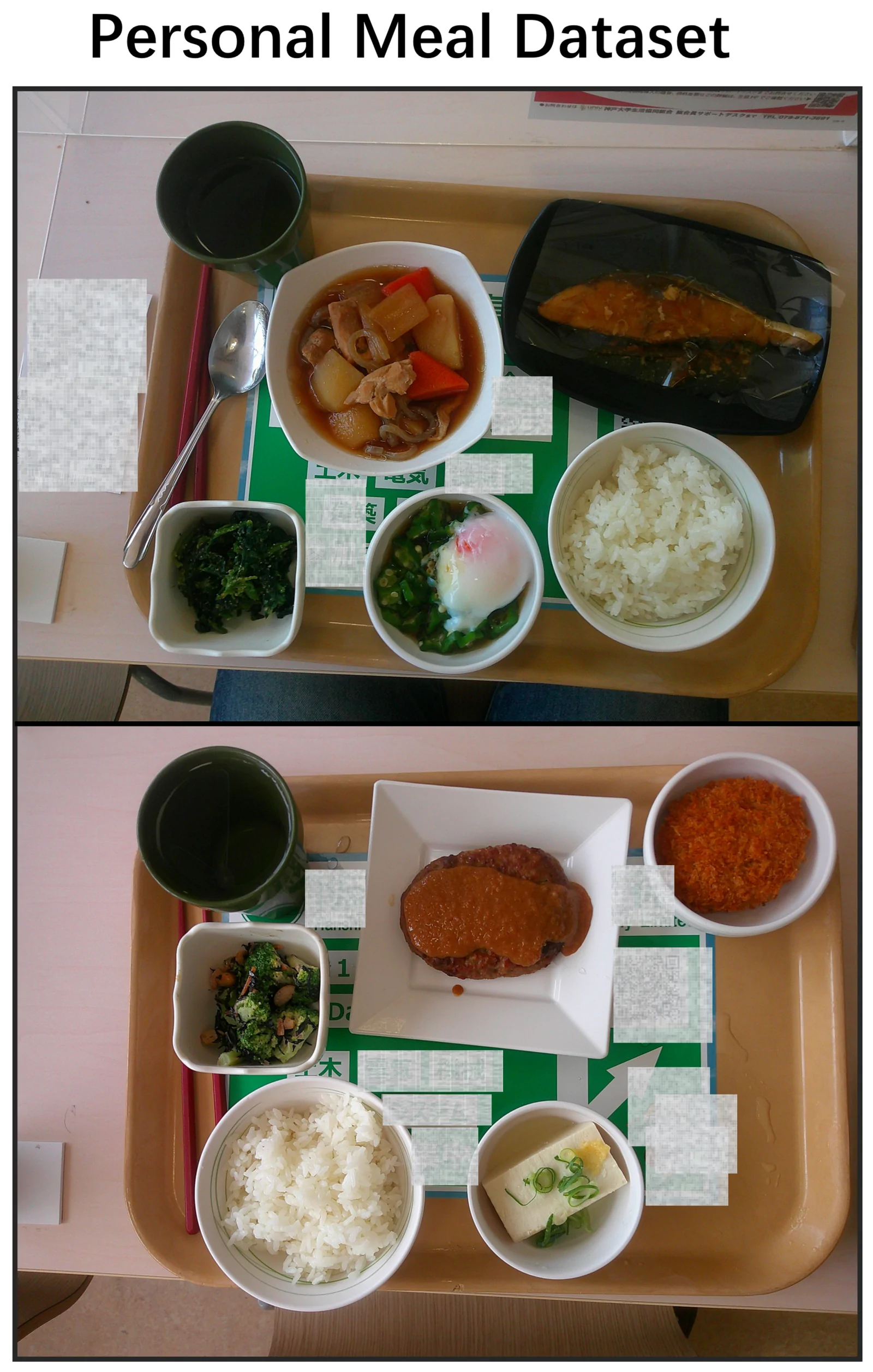
Representative non-identifiable meal images from the self-collected personal meal dataset (two examples). The two meal photographs are arranged vertically. Selected background text has been obscured, while the remaining Japanese text is part of the original dining environment and does not affect the interpretation of the meal contents. The roles of UEC FOOD 256 and FoodLogAthl-218 in this study are summarized in Table 2 and Figure 2; their original images are not reproduced here because reproduction and redistribution are governed by each dataset provider’s own usage conditions.

Figure 2 summarizes the complementary role of each of the three datasets, Table 2 lists their scale and reference characteristics side by side, and Figure 3 shows representative non-identifiable images from the self-collected personal meal dataset.

### 3.3. Personal Meal Data Preparation

The personal meal dataset was converted into an evaluation-ready crop-level dataset through a semi-automated curation procedure. Raw meal records and associated meal images were first processed using the Segment Anything Model (SAM, ViT-B), implemented using the official segment_anything Python package (version 1.0; Meta AI Research, Menlo Park, CA, USA) with the sam_vit_b_01ec64.pth checkpoint to generate food-item crops. An automatic matching stage assigned candidate UEC-256 categories and confidence scores. The resulting review table was then inspected using an interactive HTML interface that displayed each crop, the automatic match, confidence information, and manual confirmation or correction controls. A total of 2742 review-table rows were manually processed, with no unreviewed rows remaining at the end of curation. The finalization step produced 401 crop files at the recorded completion point and covered the 92 meal events used in the evaluation.

**Figure 4:**
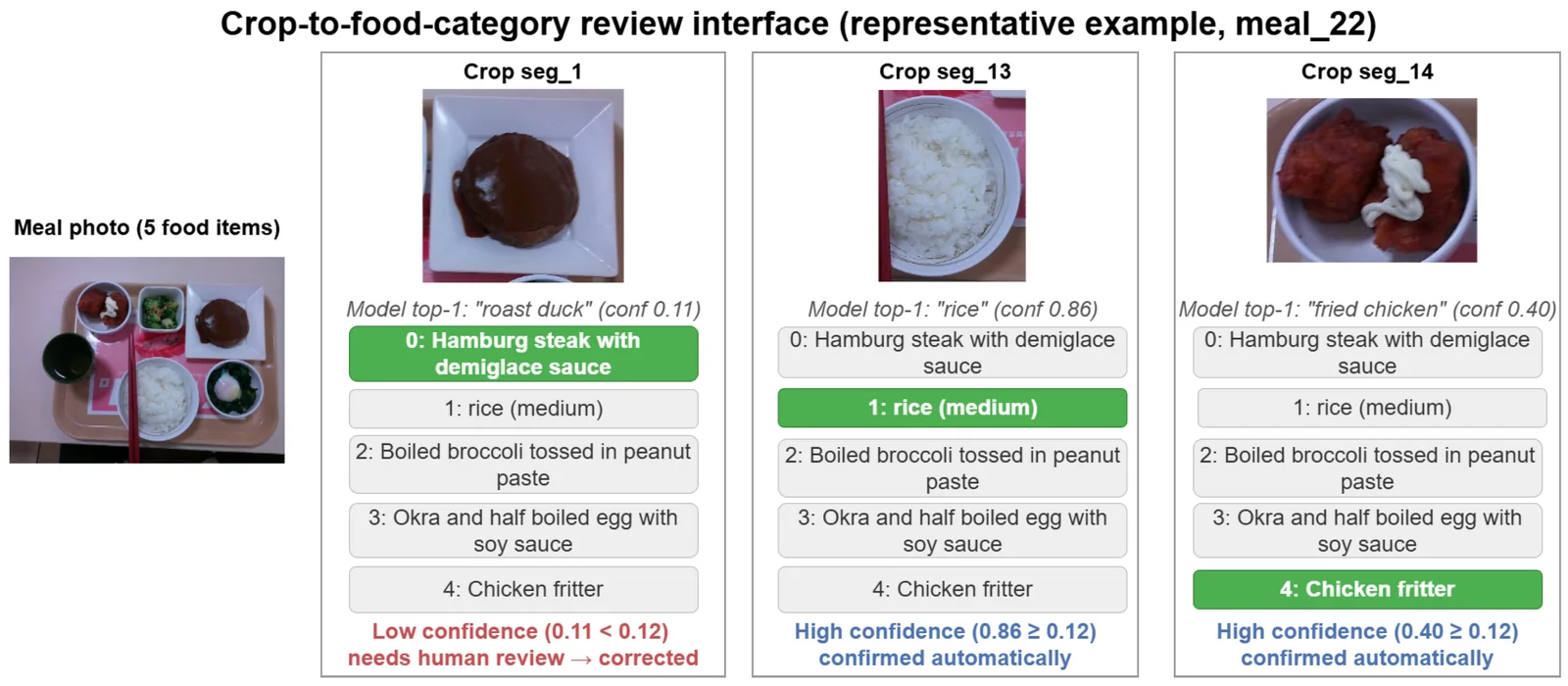
Representative example of the semi-automated crop-to-food-category curation workflow (meal_22, five food items). Each crop is shown alongside its automatic top-1 category match, confidence score, and the confidence-ranked candidate list used for manual confirmation or correction; the green-highlighted candidate indicates the final selected food category. Red text indicates low-confidence predictions requiring human review, whereas blue text indicates high-confidence predictions confirmed automatically. Gray boxes represent unselected candidate categories.

Crops with a model confidence of at least 0.12 were auto-confirmed, while crops below this threshold were routed to manual review; this threshold was chosen to be permissive (auto-confirming even fairly low-confidence matches) so that human review effort was concentrated on the crops most likely to be mismatched. The automatic matching stage achieved an overall Top-1 category accuracy of 59.2% against the manually finalized category labels. Accuracy was 74.4% in the auto-confirmed (confidence ≥0.12) subset but only 6.2% in the subset routed to manual review (confidence <0.12), motivating the use of human review for construction of the evaluation references. Figure 4 illustrates this review process using one representative meal (five food items), showing three of its crops: two auto-confirmed matches at different confidence levels (0.86 and 0.40, both above the 0.12 threshold) and one low-confidence match (0.11) that was corrected by the reviewer. This single-meal example is shown for legibility; the aggregate accuracy figures reported above summarize the full review of all 2742 crops across the 92-meal dataset. Manual confirmation and correction were used only to finalize the reference annotations for evaluation and were not fed back into the model predictions used for performance assessment.

### 3.4. CP-BayesNE Method

#### 3.4.1. Food Recognition and Probability Calibration

Food-item crops were resized and center-cropped to 224×224 pixels and normalized using ImageNet statistics. An EfficientNetV2-S backbone with a 256-class classification head was used. The classifier weights were taken from the frozen UEC-256 training run. The classification model was implemented in Python (version 3.13.9) using PyTorch (version 2.9.1+cu128) and torchvision (version 0.24.1+cu128), with CUDA 12.8, and trained on an NVIDIA GeForce RTX 5090 GPU (NVIDIA Corporation, Santa Clara, CA, USA).

Given class logits $z_{y}\left(x\right)$, temperature scaling with a scalar parameter *T* was applied as

(1) $$p_{T}\left(y|x\right)=\frac{\exp(z_{y}\left(x\right)/T)}{\sum_{j=1}^{K}\exp\left(z_{j}\left(x\right)/T\right)},$$

where K=256. The optimized temperature in the frozen calibration result was T=0.905738.

#### 3.4.2. Conformal Candidate-Set Construction

Adaptive Prediction Sets (APS) were used to construct a candidate set $C_{\alpha }\left(x\right)$ from the calibrated class probabilities [15]. This construction follows the split-conformal framework for distribution-free finite-sample coverage [47]. Under the standard split-conformal assumptions, the category-level target is

(2) $$\mathrm{Pr}\left\{Y\in C_{\alpha }\left(X\right)\right\}\geq 1-\alpha .$$

The main experiments used α=0.10. A finite-sample conformal quantile correction was applied when estimating the calibration threshold.

Regularized Adaptive Prediction Sets (RAPS) [16] were additionally evaluated to improve candidate-set efficiency, addressing the coverage-versus-set-size trade-off inherent to adaptive prediction sets [48]. RAPS shares the same marginal coverage target as APS (Equation (2)) but constructs the candidate set differently: writing $\pi_{\left(1\right)}\geq \pi_{\left(2\right)}\geq \cdots \geq \pi_{\left(K\right)}$ for the calibrated probabilities sorted in decreasing order and o(y) for the rank of class *y*, the RAPS nonconformity score adds a regularization penalty to the cumulative-probability score used by APS,

(3) $$E\left(x,y\right)=\sum_{j=1}^{o(y)}\pi_{\left(j\right)}\;+\;\lambda \max\left(o\left(y\right)-k_{\mathrm{reg}},\;0\right),$$

so that classes ranked beyond $k_{\mathrm{reg}}$ incur an additional cost λ per rank, discouraging low-probability classes from being retained in $C_{\alpha }\left(x\right)$ purely to reach the coverage target. The regularization parameter λ was selected on the UEC-256 validation split from the candidate grid {0,0.001,0.005,0.01,0.02,0.05,0.1,0.2,0.5}, and the selected model was evaluated on the independent test split. For α=0.10, the frozen configuration used λ=0.5 and $k_{\mathrm{reg}}=5$. The main downstream personalization experiments (Section 4.2 and Section 4.3) use APS candidate sets throughout for consistency with the primary experimental design (Table 3); Section 4.1 additionally reports a direct APS-versus-RAPS comparison on FoodLogAthl-218 nutrient-density MAE.

RAPS was not re-evaluated on the 92-meal personal dataset.

#### 3.4.3. Prior-Corrected Dietary Personalization

For user *u*, let $c_{u,y}$ denote the number of historical occurrences of category *y*, and let $N_{u}=\sum_{y}c_{u,y}$. A Laplace-smoothed personal dietary prior is defined as

(4) $$\pi_{u}\left(y\right)=\frac{c_{u,y}+\beta }{N_{u}+K\beta },\;\beta =1.$$

The calibrated discriminative posterior $p_{T}\left(y|x\right)$ already reflects the class prior of the UEC-256 training distribution. To avoid directly multiplying this posterior by an additional user prior without correcting for the training prior, CP-BayesNE uses a prior-replacement form. Let $\pi_{\mathrm{tr}}\left(y\right)$ denote the smoothed UEC-256 training prior. The personalized score is

(5) $${\tilde{p}}_{u}\left(y|x\right)\propto \frac{p_{T}\left(y|x\right)}{\pi_{\mathrm{tr}}\left(y\right)}\pi_{u}\left(y\right),\;y\in C_{\alpha }\left(x\right),$$

followed by normalization within the conformal candidate set. This operation can be interpreted as prior-corrected adaptation under a prior-shift assumption [20,21,22]; it should not be interpreted as evidence that the full image-generation process is governed by a specified generative Bayesian model.

#### 3.4.4. Nutrient Estimation

Let $n_{k}\left(y\right)$ denote the per-100 g reference value of nutrient *k* for category *y*, where *k* corresponds to energy, protein, fat, or carbohydrate. The personalized nutrient-density estimate is

(6) $${\hat{n}}_{u,k}\left(x\right)=\sum_{y\in C_{\alpha }\left(x\right)}w_{u}\left(y|x\right)n_{k}\left(y\right),$$

where $w_{u}\left(y|x\right)$ denotes the normalized prior-corrected posterior weight within the candidate set.

For analyses that use an assumed standard portion g(y) in grams, the category-level portion-scaled value is

(7) $$e_{k}\left(y\right)=n_{k}\left(y\right)\frac{g(y)}{100},$$

and the posterior-weighted estimate is obtained by replacing $n_{k}\left(y\right)$ with $e_{k}\left(y\right)$ in Equation (6). Because the available datasets do not contain independently measured consumed weights for all items, such values are described as portion-scaled nutrient estimates rather than true weighed intake.

#### 3.4.5. Nutrient-Value Uncertainty Calibration

Category-level conformal coverage does not automatically imply coverage for derived nutrient values. We therefore calibrated nutrient-value uncertainty separately, following normalized/studentized nonconformity scoring [49] and locally adaptive conformal regression [47,50]. For each nutrient *k*, let ${\hat{z}}_{i,k}$ and ${\hat{\sigma }}_{i,k}$ denote the posterior-weighted mean and weighted standard deviation for sample *i*, and let $z_{i,k}$ denote the available nutrient reference. A studentized nonconformity score was computed as

(8) $$r_{i,k}=\frac{|z_{i,k}-{\hat{z}}_{i,k}|}{\max({\hat{\sigma }}_{i,k},\epsilon )}.$$

A finite-sample conformal quantile $q_{k}$ was estimated from held-out residuals. The resulting nutrient interval was

(9) $$I_{i,k}=\left[{\hat{z}}_{i,k}-q_{k}{\hat{\sigma }}_{i,k},\;{\hat{z}}_{i,k}+q_{k}{\hat{\sigma }}_{i,k}\right].$$

Calibration was performed separately for each nutrient. Five-fold cross-validation was used for the personal dataset with folds defined at the meal level, and five-fold cross-validation was used for FoodLogAthl-218 with folds defined at the user level.

### 3.5. Secondary Adaptive Refinement

A secondary adaptive refinement was evaluated to reduce errors when the personal dietary prior strongly changed the top category within the conformal candidate set. For each crop, a personalized posterior and a training-prior-corrected non-personalized posterior were computed over the same candidate set. The agreement was defined by the equality of their highest posterior candidate. When the two decisions agreed, all nutrient estimates used the personalized posterior. When they disagreed, energy and carbohydrate retained the personalized estimate, whereas protein and fat reverted to the non-personalized estimate.

This rule is treated as a secondary empirical refinement rather than a universally derived decision rule. The nutrient-specific choice of protein and fat was identified after diagnostic analysis of the personal dataset and is therefore potentially data-dependent. The same fixed rule was subsequently transferred without modification to FoodLogAthl-218, where it also reduced aggregate MAE. This independent transfer is supportive but does not remove the methodological limitation associated with the original rule selection.

### 3.6. Experimental Design and Statistical Analysis

The experiments were organized around four questions: recognition uncertainty, user-level personalization, history-size sensitivity, and real-meal validation. The principal comparison for population-level personalization used the prior-corrected non-personalized condition (A) and the prior-corrected personalized condition (B). The adaptive condition (C) was treated as a secondary refinement. RAPS was evaluated separately for category-set efficiency. Table 3 summarizes these conditions.

Nutrient-estimation error was summarized using mean absolute error (MAE). For FoodLogAthl-218, per-user MAEs were calculated and then macro-averaged across users so that the statistical unit was the user rather than the crop. Paired user-level comparisons used the Wilcoxon signed-rank test. For condition B, the confirmatory analysis also reported the number of users with improved and worsened MAE.

## 4. Results

The following four subsections are organized around the four objectives introduced in Section 1, in the same order: whether conformal prediction can represent food-category uncertainty with practical candidate-set efficiency (Section 4.1); whether prior-corrected dietary personalization improves nutrient-density estimation at the user level (Section 4.2); whether this benefit depends on the amount of dietary history available (Section 4.3); and whether downstream nutrient uncertainty requires calibration separate from category-level conformal prediction (Section 4.4).

### 4.1. Recognition Uncertainty and Conformal Prediction

This subsection addresses the first objective: whether the calibrated classifier and its conformal candidate sets represent food-category uncertainty in a way that is both statistically valid and practically usable, rather than either collapsing to an unreliable Top-1 decision or requiring an impractically large candidate set. Recognition performance and calibration quality were measured on the frozen UEC-256 test split (Section 3.4.1); APS and RAPS candidate-set coverage and size were then measured at α=0.10 using the λ and $k_{\mathrm{reg}}$ values selected on the validation split (Section 3.4.2). The EfficientNetV2-S classifier achieved a Top-1 accuracy of 78.01% and a Top-5 accuracy of 93.67% on the frozen UEC-256 test split. Temperature scaling reduced expected calibration error (ECE) from 0.11238 to 0.04345, a relative reduction of 61.34%, with an optimized temperature of 0.905738 (Table 4).

**Figure 5:**
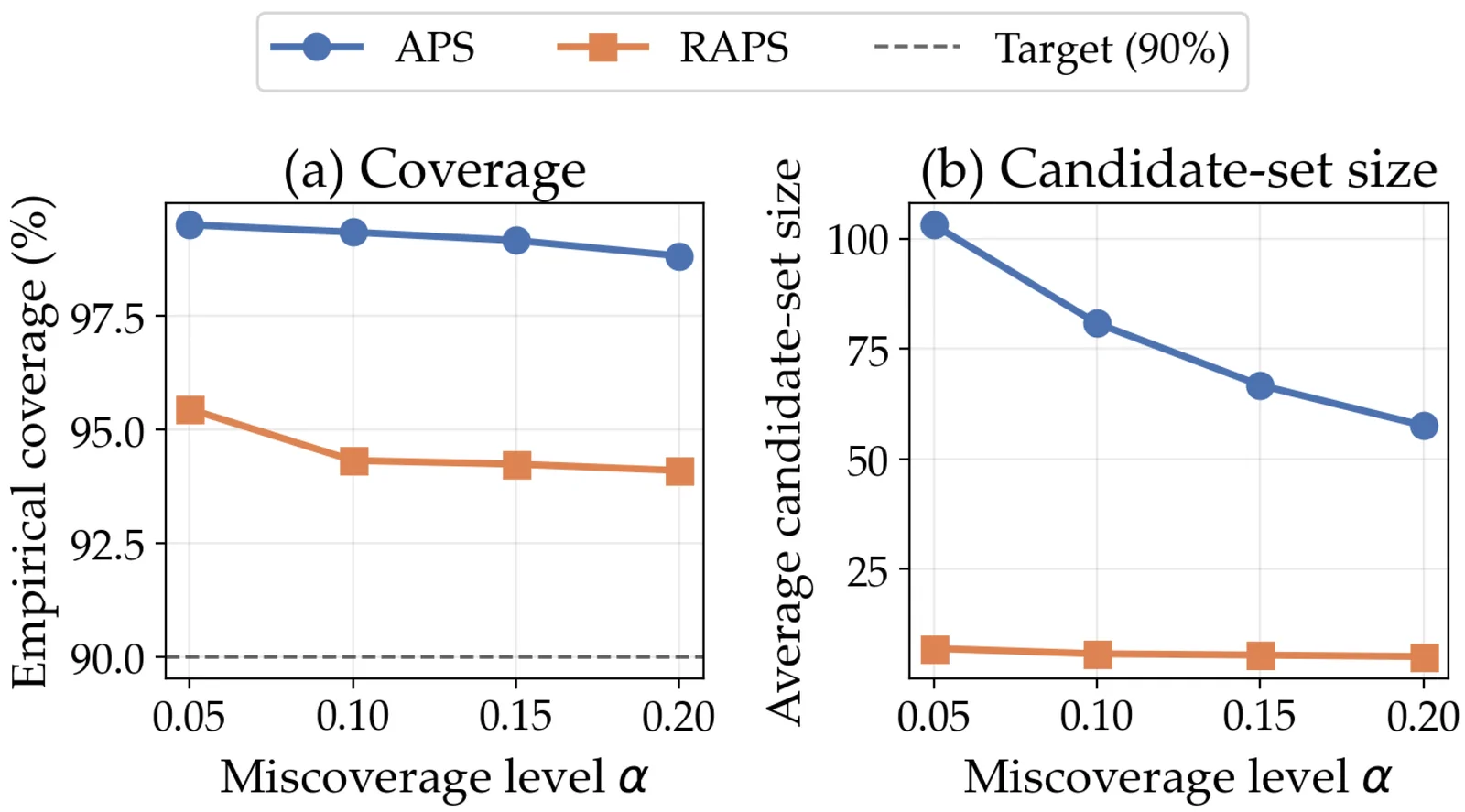
Comparison of APS and RAPS in category-level empirical coverage and candidate-set efficiency. (**a**) Empirical category-level coverage of APS and RAPS across different miscoverage levels α, with the dashed line indicating the 90% coverage target; (**b**) average candidate-set size of APS and RAPS across different miscoverage levels α.

APS produced highly conservative candidate sets. At α=0.10, empirical category coverage was 99.35%, but the average candidate-set size was 80.78 classes. RAPS substantially improved set efficiency. With λ=0.5 and $k_{\mathrm{reg}}=5$, empirical coverage was 94.32% and the average candidate-set size was 5.77, corresponding to a 92.85% reduction relative to APS (Figure 5). Taken together, these results answer the first objective in the affirmative: a moderate Top-1 accuracy combined with substantially higher Top-5 accuracy indicates that a plausible alternative category is usually available even when the top prediction is wrong, and RAPS shows that this uncertainty can be represented with a candidate set small enough for downstream use, rather than the 80-class set APS alone would require.

### 4.2. Population-Level Personalized Nutrient Estimation

This subsection addresses the second objective: whether the prior-corrected dietary-history personalization introduced in Section 3.4.3 improves nutrient-density estimation at the user level and whether that improvement is a broad, statistically consistent tendency across users rather than an artifact of a few individuals. This was tested on FoodLogAthl-218 across 51 independent users, comparing prior-corrected personalization (condition B) against the prior-corrected non-personalized baseline (condition A) on macro-averaged nutrient-density MAE, with user-level Wilcoxon signed-rank tests as the confirmatory check. Across 51 FoodLogAthl-218 users, prior-corrected personalization reduced macro-averaged nutrient-density MAE for all four targets relative to the prior-corrected non-personalized baseline. Energy MAE decreased from 37.049 to 35.323 kcal/100 g (4.7%), protein from 2.576 to 2.454 g/100 g (4.7%), fat from 3.167 to 3.041 g/100 g (4.0%), and carbohydrate from 6.810 to 6.364 g/100 g (6.6%). These values represent the corrected evaluation and replace previously reported larger improvements from the earlier direct-prior implementation (Table 5).

The secondary adaptive condition further reduced the aggregate MAE to 33.396 kcal/100 g for energy, 2.329 g/100 g for protein, 2.915 g/100 g for fat, and 5.726 g/100 g for carbohydrate. The corresponding nutrient-interval empirical coverage values were 0.900, 0.899, 0.901, and 0.902, respectively.

Because RAPS substantially reduced candidate-set size relative to APS (Section 4.1), we additionally checked whether this reduction translated into a downstream difference in nutrient-density estimation, rather than only a category-level efficiency gain. The prior-corrected personalization pipeline (condition B) was rerun on FoodLogAthl-218 with the conformal candidate set constructed by RAPS (λ=0.5, $k_{\mathrm{reg}}=5$, the same frozen configuration as in Section 4.1) instead of APS, holding the Bayesian personal-prior update and all other pipeline components fixed. Replacing APS with RAPS reduced nutrient-density MAE for all four targets under both the non-personalized and personalized conditions (Table 6). For condition B, MAE decreased by 2.3% for energy, 4.1% for protein, 7.1% for fat, and 7.7% for carbohydrate relative to the APS-based result in Table 5. This indicates that RAPS’s smaller candidate set did not trade downstream accuracy for category-set efficiency; on this dataset, the more compact candidate set was associated with equal or better nutrient-density estimates. RAPS was not, however, re-evaluated on the 92-meal personal dataset, so this comparison is limited to the population-level FoodLogAthl-218 setting.

**Figure 6:**
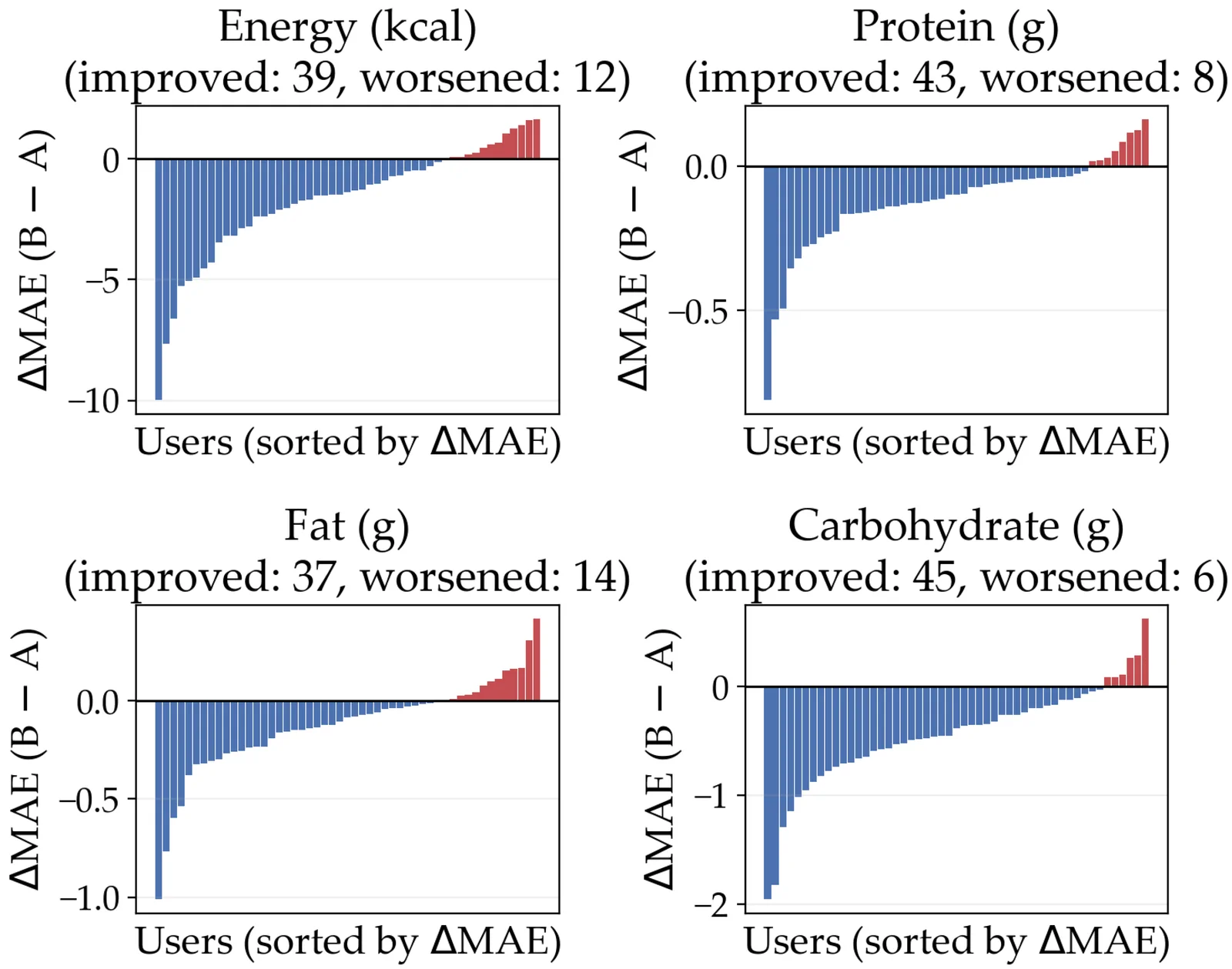
User-level paired changes in nutrient-density MAE after prior-corrected dietary personalization. The change in MAE (ΔMAE) is calculated as Condition B minus Condition A. Blue bars indicate reduced MAE (improvement), whereas red bars indicate increased MAE (worsening). The horizontal black line represents no change (ΔMAE=0). Users are sorted by their individual MAE changes within each nutrient.

For the confirmatory B-versus-A comparison, personalization improved energy MAE in 39 of 51 users, protein in 43, fat in 37, and carbohydrate in 45. Two-sided Wilcoxon signed-rank tests were significant for all four nutrient targets and remained significant after Holm correction for simultaneous testing across the four nutrients (Table 7; per-user paired changes shown in Figure 6). These statistics refer to condition B, not the final adaptive condition C. Together, these results answer the second objective in the affirmative for the population as a whole: the improvement is not driven by a small subset of users, but the modest effect size (4.0–6.6% MAE reduction) also indicates that personalization refines an already-reasonable population-level estimate rather than correcting a large systematic error.

### 4.3. Effect of Dietary History

**Figure 7:**
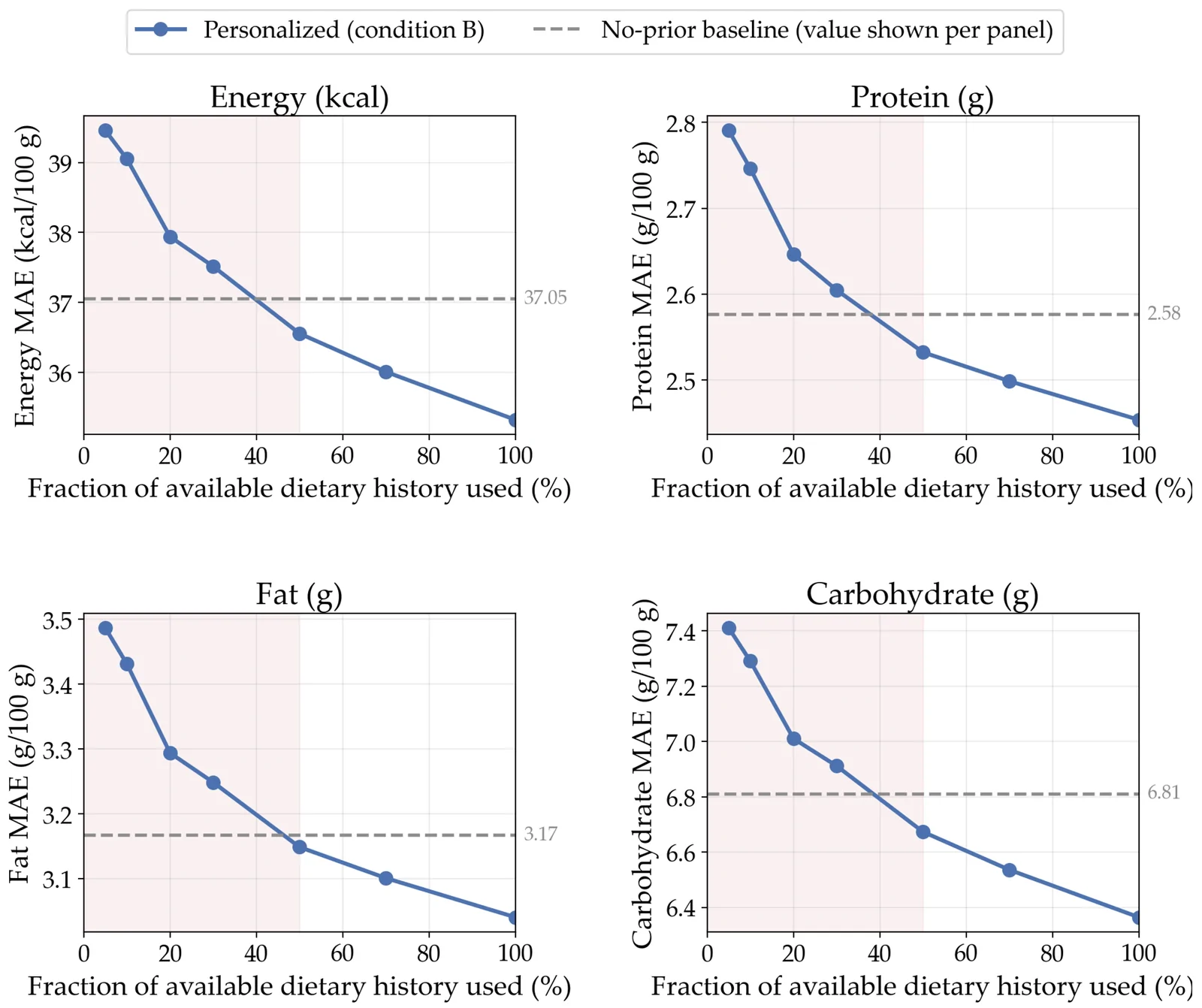
History-size sensitivity of prior-corrected personalization across all four nutrient targets in FoodLogAthl-218. The shaded region indicates the range in which no more than 50% of the available dietary history is used.

This subsection addresses the third objective: whether the population-level benefit established in Section 4.2 holds regardless of how much dietary history a user has accumulated, or whether it instead requires a minimum amount of history to emerge. This was tested by recomputing the prior-corrected personalization result for all four nutrient targets at successive fractions (5% to 100%) of each FoodLogAthl-218 user’s available training history, holding all other pipeline components fixed. The benefit of personalization depended on the amount of user history available, and this pattern was consistent across all four nutrients. For energy, the no-prior baseline MAE was 37.05 kcal/100 g; the personalized MAE was 39.46 kcal/100 g with only 5% of the available history and remained above the baseline at 10% (39.05), 20% (37.94), and 30% (37.51), before first outperforming the baseline between 30% and 50% of accumulated history, reaching 36.56 at 50%, 36.01 at 70%, and 35.32 at 100%. Protein (baseline 2.58 g/100 g), fat (baseline 3.17 g/100 g), and carbohydrate (baseline 6.81 g/100 g) each showed the same qualitative pattern: personalized MAE started above the no-prior baseline at 5% of history (2.79, 3.49, and 7.41 g/100 g, respectively), remained above baseline through 30% of history, and crossed below baseline between 30% and 50% of accumulated history, reaching 2.45, 3.04, and 6.36 g/100 g at 100% of history (Figure 7).

These results answer the third objective: the benefit is not unconditional. A cold-start regime exists in which insufficient dietary history makes personalization unhelpful or actively harmful, and only after roughly 30–50% of the available history had accumulated did personalization begin to outperform the no-prior baseline. This cold-start threshold was consistent across all four nutrient targets under condition B; the corresponding curve for condition C was not available in the frozen result set.

### 4.4. Real-Meal Validation

This analysis was intended as a single-participant feasibility evaluation of the end-to-end pipeline, not as a population-level validation of absolute dietary intake; the population-level evidence for the personalization mechanism itself is established separately in Section 4.2 using 51 independent FoodLogAthl-218 users. This subsection addresses the fourth objective: whether downstream nutrient-value uncertainty genuinely requires calibration separate from category-level conformal prediction, as argued in Section 1, or whether a valid category-level candidate set is in practice already enough to yield trustworthy nutrient values. This was tested end-to-end on the 92-meal personal dataset, comparing point-estimate accuracy (meal-level MAE) against the non-personalized baseline, and separately checking whether the independently calibrated nutrient-value intervals (Section 3.4.5) achieved their target coverage and did so with a practically useful width. The personal dataset provided an end-to-end evaluation across 92 real multi-item meals. Relative to the non-personalized condition using the personal-portion configuration, the final adaptive condition reduced energy MAE from 323.10 to 313.44 kcal (3.0%), protein MAE from 14.27 to 14.14 g (0.9%), and carbohydrate MAE from 34.06 to 27.03 g (20.6%). Fat MAE increased from 14.64 to 15.12 g (3.3%). The fat result is therefore reported as a negative result rather than as an improvement (Table 8).

**Figure 8:**
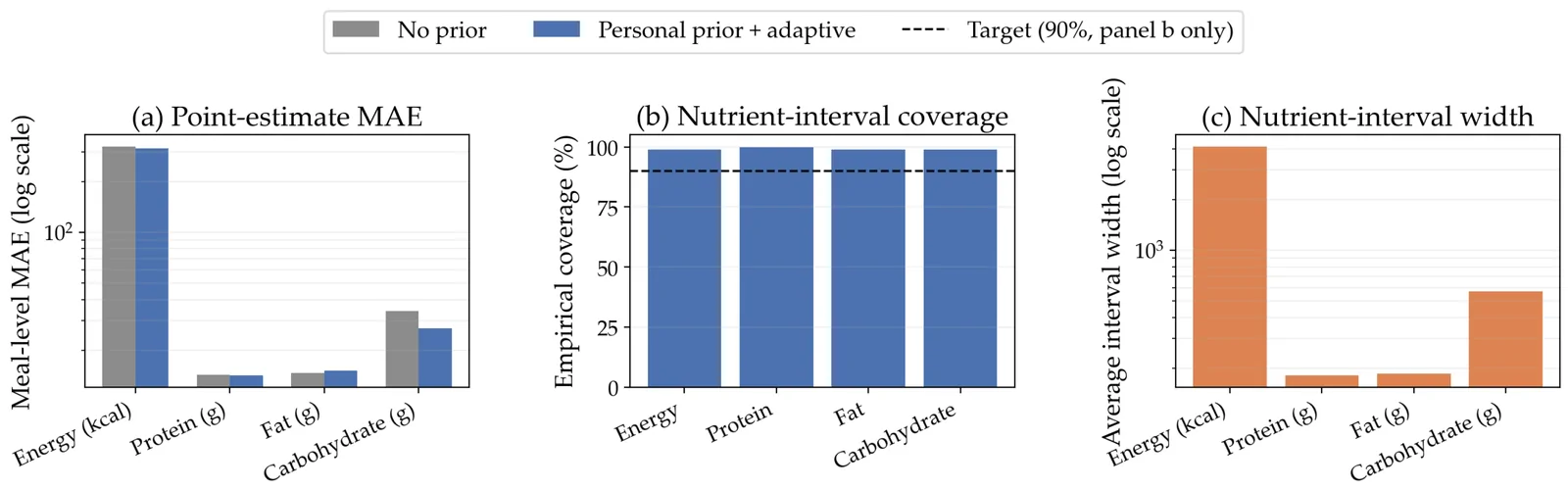
Point-estimation error and calibrated nutrient-interval performance in the 92-meal personal dataset. (**a**) Meal-level mean absolute error (MAE) for energy, protein, fat, and carbohydrate under the no-prior and personal-prior-plus-adaptive conditions (logarithmic scale); (**b**) empirical coverage of the calibrated nutrient intervals for the four nutrient targets, with the dashed line indicating the 90% target coverage; (**c**) average width of the calibrated nutrient intervals for the four nutrient targets (logarithmic scale).

Separately calibrated nutrient intervals achieved empirical coverage of 98.9% for energy, 100% for protein, 98.9% for fat, and 98.9% for carbohydrate. However, this high coverage was accompanied by conservative average interval widths. In the frozen evaluation output, the average widths were 4141.7 kcal for energy, 181.0 g for protein, 186.1 g for fat, and 570.1 g for carbohydrate (Figure 8). These values indicate that restoring coverage alone does not establish interval efficiency or practical usefulness. Taken together with the point-estimate results above, this answers the fourth objective in the affirmative: a category-level candidate set with valid coverage does not automatically translate into a narrow or immediately useful nutrient-value interval, confirming that the separate calibration step introduced in Section 3.4.5 addresses a real gap rather than a redundant one. These wide intervals indicate that the current residual-based nutrient-interval calibration prioritizes coverage over efficiency, and further methodological refinement is needed before the intervals can be used for practical dietary feedback.

**Figure 9:**
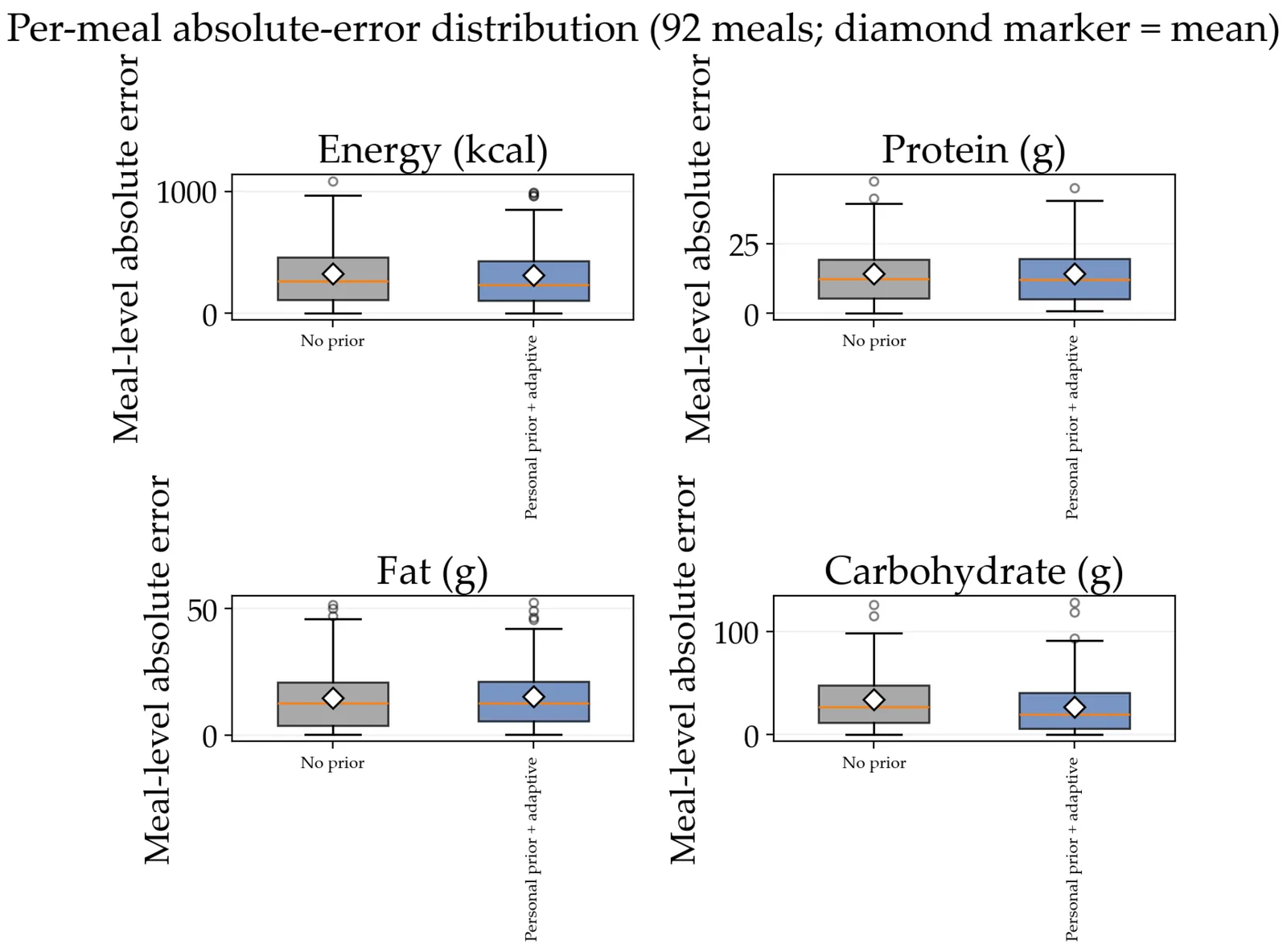
Distribution of meal-level absolute error across all 92 meals, by nutrient and condition. Boxes show the interquartile range and median; diamonds mark the mean (matching Table 8); circles mark individual meals beyond the whiskers. Gray boxes represent the no-prior condition (Condition A), whereas blue boxes represent the personal-prior-plus-adaptive condition (Condition C).

The aggregate MAE values above summarize central tendency only and do not by themselves indicate whether errors were consistently modest or dominated by a small number of large misses. Figure 9 shows the full per-meal absolute-error distribution for both conditions: for all four nutrients, the median error was lower than the mean, and a small number of meals with substantially larger errors are visible as distributional outliers. This pattern is consistent with the base-classifier misrecognition ceiling discussed in Section 5.5, concentrating error in a minority of meals, rather than indicating a general lack of reliability across the dataset.

To replace the earlier preliminary 36-meal domain-shift check with an analysis based on the final evaluation dataset, we evaluated category-level coverage on the full 92-meal dataset under cross-domain calibration (the UEC-256-calibrated threshold applied directly to personal-meal crops) and same-domain calibration, in which one half of the personal meals was used exclusively for conformal calibration, and the disjoint remaining half was used for coverage evaluation, with all crops from a given meal kept within the same split. At α=0.10, cross-domain coverage was 92.75% and same-domain coverage was 93.81% on this disjoint evaluation half, both meeting the nominal 90% target, with a similar pattern previously observed on FoodLogAthl-218 (90.84% cross-domain, 92.73% same-domain; Table 9). Category-level coverage was therefore preserved under domain shift in both independent real-photo datasets. However, the average candidate-set size needed to reach this coverage increased to approximately 101–103 classes on the personal dataset and 106–118 classes on FoodLogAthl-218, compared with 80.78 classes on the curated UEC-256 test split (Table 4). This increase reflects the practical cost of distributional shift anticipated in Section 1: coverage validity is preserved, but candidate-set efficiency is reduced on real, uncurated photographs relative to the benchmark domain. This check used the standard/APS-style conformal construction only; whether the RAPS efficiency gain reported in Section 4.1 also transfers under domain shift was not evaluated and is noted as a direction for future work.

## 5. Discussion

### 5.1. Principal Findings

This study evaluated CP-BayesNE as an uncertainty-aware personalized dietary-assessment framework rather than as a new food-classification architecture. Four findings are central. First, the base food classifier achieved moderate Top-1 accuracy but substantially higher Top-5 accuracy, indicating that plausible alternative food categories frequently remain available even when the top prediction is wrong. Second, APS provided conservative category-level coverage but produced impractically large candidate sets, whereas RAPS reduced the average set size by more than 90% while maintaining empirical coverage above the nominal 90% target at the main operating point. Third, prior-corrected dietary-history personalization produced modest but statistically consistent reductions in nutrient-density MAE across 51 FoodLogAthl-218 users. Fourth, real-meal evaluation showed that downstream nutrient uncertainty remained a distinct challenge: category-level conformal validity did not automatically imply narrow or practically efficient nutrient intervals.

### 5.2. Conformal Uncertainty and Candidate-Set Efficiency

The contrast between APS and RAPS illustrates the difference between coverage and efficiency. APS exceeded the nominal target by a wide margin at α=0.10, but the resulting average candidate set contained more than 80 classes. Such a set may satisfy a statistical coverage objective while remaining too broad for practical nutrient inference or human interpretation. RAPS substantially reduced the candidate-set size while maintaining high empirical coverage. This result supports the use of regularized conformal sets when the downstream task benefits from compact candidate sets. A direct comparison on FoodLogAthl-218 (Table 6) further showed that replacing APS with RAPS reduced condition-B nutrient-density MAE for all four targets, indicating that the more compact candidate set did not come at the cost of downstream accuracy on these datasets. This comparison was not repeated on the 92-meal personal dataset, so the finding should be read as evidence from the population-level FoodLogAthl-218 setting specifically, not as a claim generalized across all downstream evaluations in this study. Because APS was the predefined candidate-set construction used throughout the primary 92-meal pipeline (Table 3), introducing RAPS only for this dataset at the revision stage would constitute an additional secondary analysis rather than part of the original primary design; we therefore did not re-run the 92-meal pipeline under RAPS-constructed candidate sets and identify this as a concrete direction for future work. Because the APS-based analysis was used as the predefined primary personalization comparison, RAPS-B was treated as a secondary sensitivity analysis rather than a replacement for the primary design; future implementations should preferentially use RAPS or other efficiency-controlled conformal sets given the results above.

### 5.3. Dietary-History Personalization and Cold Start

The FoodLogAthl-218 results support the use of personal dietary history as an additional source of information when the visual evidence remains ambiguous. The prior-corrected comparison is important because the discriminative classifier posterior already reflects the class prevalence of the training set, consistent with the label/prior-shift framework [20,21]. Dividing out this prior before introducing the user-specific prior avoids the earlier direct-multiplication formulation that could inflate the contribution of prior information.

The magnitude of improvement was modest rather than dramatic, but the direction was consistent across all four nutrient-density targets and across a majority of users. The history-size analysis also showed that personalization should not be assumed to help immediately. With very limited history, energy MAE was worse than the no-prior baseline, and improvement emerged only after a substantial amount of user history had accumulated. This cold-start behavior is practically important for longitudinal systems and argues for adaptive activation or cautious weighting of personalization during early use. Based on the history-size sensitivity results in Section 4.3, where all four nutrient targets first showed consistent improvement over the no-prior baseline between 30% and 50% of each user’s accumulated dietary history, we propose an evaluation-derived conservative activation criterion: personalization should remain disabled, defaulting to the non-personalized estimate, until a user has accumulated at least 50% of a stable reference history window, the earliest point at which all four nutrient targets showed consistent improvement in our data. This criterion is derived from the present population-level evaluation rather than already validated as a deployable threshold: it is defined relative to each user’s own eventual history size here and would need to be re-expressed as an absolute minimum record count, calibrated on a representative deployment population, before it could be applied as a fixed rule in an operational system.

The nutrient-specific adaptive refinement provided additional aggregate improvements in the independent FoodLogAthl-218 evaluation. However, because the rule determining which nutrient targets fall back to the non-personalized estimate was derived after diagnostic analysis of the personal dataset, the refinement should be interpreted as secondary evidence rather than as a universal decision rule.

### 5.4. From Recognition Uncertainty to Nutrient Uncertainty

A major methodological lesson from the present analysis is that food-category uncertainty and nutrient-value uncertainty are not interchangeable. A conformal candidate set addresses the event that the correct food category is contained in the candidate set. Nutrient estimation, however, depends on how probability mass is distributed across candidate categories, on the nutrient reference associated with each category, on portion assumptions, and on aggregation across meal items [23,24]. It is therefore possible for category-level coverage to remain adequate while nutrient intervals substantially under-cover.

The studentized-residual calibration restored empirical nutrient-value coverage in both FoodLogAthl-218 and the personal dataset. However, the personal meal results also demonstrate a second requirement: interval validity should be evaluated together with interval efficiency. Very wide intervals can achieve high coverage while offering limited practical information, echoing the coverage-width trade-off documented for adaptive conformal regression [47,50]. A likely contributing mechanism is the small number of residuals available for quantile estimation in each calibration fold: with five-fold cross-validation at the meal level, each training fold contains on the order of 70 meals, so the order statistic required for a 90% empirical quantile sits close to the largest observed residual in that fold, making $q_{k}$ highly sensitive to a small number of outlier meals. Because interval width in Equation (9) scales directly with $q_{k}$, this calibration-sample-size effect, rather than classifier training-sample size or per-image estimation noise, is the primary candidate explanation for the observed width inflation. Narrowing these intervals without sacrificing coverage would likely require larger calibration cohorts, locally adaptive quantile estimation that pools information across similar meals rather than estimating $q_{k}$ per fold independently, or a learned, meal-composition-aware variance model in place of the candidate-set-internal weighted standard deviation ${\hat{\sigma }}_{i,k}$. Future refinement should therefore consider both coverage and width, ideally using stronger portion reference [8] and broader real-meal cohorts. This negative result is important because it prevents overinterpretation of category-level conformal validity as a guarantee of practically useful nutrient-level feedback.

### 5.5. Strengths, Limitations, and Future Directions

A strength of the study is the use of complementary validation levels. UEC-256 supports controlled classifier and conformal evaluation; FoodLogAthl-218 provides user-level population evidence; and the 92-meal personal dataset tests the full multi-item pipeline under real meal-recording conditions. The explicit separation between nutrient-density evaluation and meal-level declared-value evaluation also reduces the risk of mixing reference targets with different evidential strength.

Several limitations remain. First, neither FoodLogAthl-218 nor the personal dataset provides a gold-standard weighed-intake reference. FoodLogAthl-218 lacks portion annotations entirely, whereas the personal dataset uses dish- or product-level declared nutrient values. Second, the real-meal dataset represents a single individual and therefore cannot establish population-level absolute intake accuracy. Third, fat MAE remained slightly worse than the non-personalized baseline in the personal dataset. Fat estimation may be particularly sensitive to preparation-level variation because relatively small amounts of added oil, dressing, or sauce can materially change the fat content of a dish without producing a correspondingly obvious visual change. Consequently, two visually similar dishes assigned to the same food category may still differ substantially in fat content depending on the cooking method and added ingredients. Because the present datasets do not contain recipe-level oil, sauce, or preparation metadata, this explanation remains a plausible hypothesis rather than an empirically verified mechanism. Fourth, the adaptive protein/fat fallback rule was identified from the same personal dataset later used for reporting, which introduces a risk of data-specific overfitting despite its supportive transfer to FoodLogAthl-218. We treat the independent replication on FoodLogAthl-218’s 51 users as out-of-sample evidence that the qualitative direction of this rule is unlikely to be a dataset-specific artifact; however, the exact effect sizes reported on the personal dataset itself remain a post hoc, and therefore potentially optimistic, estimate that this independent replication does not retroactively correct. Fifth, the base classifier remains an important performance ceiling; incorrect food recognition can propagate large errors into downstream nutrient estimates. Sixth, the domain-shift check for personal meals was performed on the final 92-meal dataset, superseding the earlier preliminary analysis based on 36 meals (Section 4.4; Table 9). The final analysis confirmed that category-level coverage was preserved under domain shift, although at the cost of a larger candidate-set size than on the curated UEC-256 benchmark. Finally, all three datasets were collected in a Japanese dietary context (UEC-256 and the personal dataset) or from users of a Japanese dietary-logging application (FoodLogAthl-218), so the food-composition references, the trained classifier’s category space, and the personalization and nutrient-estimation results reported here are specific to this cuisine and food-presentation style. Western European or North American meals typically differ in dish composition, portion structure, and plating and would likely require a different or extended food-category taxonomy and a recalibrated classifier before the framework’s category-level and nutrient-density conclusions could be expected to transfer; we did not evaluate this generalization and identified it as an important direction for future work.

Future work should evaluate the framework using multiple independent real-meal participants with weighed or otherwise stronger nutrient references, improve food recognition under domain shift, develop data-independent strategies for selective personalization, and evaluate longitudinal adaptation using genuinely time-resolved protocols. In the broader research program, the dietary-assessment outputs may subsequently be integrated with longitudinal physical- or muscle-function measurements, but such relationships are outside the scope of the present study.

From a nutrition-assessment perspective, the proposed framework does not replace weighed food records or professional dietary assessment. Rather, it provides a lower-burden computational layer for repeated dietary monitoring when exact portion information is unavailable. Such a layer may be useful for identifying longitudinal dietary patterns, prioritizing records for human review, and quantifying uncertainty in AI-assisted dietary assessment.

## 6. Conclusions

CP-BayesNE provides a structured, AI-driven approach to precision nutrition by combining calibrated food recognition, conformal candidate sets, training-prior-corrected dietary-history personalization, and separately calibrated nutrient-value uncertainty for personalized nutrient estimation from food images. On UEC-256, temperature scaling substantially improved probability calibration, and RAPS reduced the average conformal candidate-set size from 80.78 to 5.77 at α=0.10 while maintaining 94.32% empirical category coverage. Across 51 FoodLogAthl-218 users, prior-corrected personalization reduced nutrient-density MAE by 4.0–6.6% for energy, protein, fat, and carbohydrate. The history-size analysis showed a clear cold-start effect, with personalization becoming beneficial only after sufficient user history had accumulated. A 92-meal personal evaluation further demonstrated the feasibility of the end-to-end pipeline, with improvements for energy, protein, and carbohydrate but a slight deterioration for fat.

The results also identify an important boundary of conformal dietary inference: category-level coverage does not directly guarantee reliable nutrient-value intervals. Separate residual-based calibration restored empirical nutrient coverage, but the real-meal intervals remained conservative, demonstrating the need to evaluate interval efficiency in addition to nominal coverage. Overall, the findings support uncertainty-aware and history-informed dietary assessment while avoiding claims of direct true-intake accuracy. Validation with stronger portion references and multiple independent real-meal participants is required before the framework can support population-level claims about absolute dietary intake.

## Figures and Tables

**Table 1:** Comparison of representative related work and the present study.

Related Work Category	Representative Studies	Main Focus	Remaining Gap for This Study’s Objective	Position Relative to the Present Study
Digital and precision dietary assessment	Cuparencu et al., 2026 [25]; Joyce et al., 2026 [26]; Abeltino et al., 2025 [28]; Phalle and Gokhale, 2025 [29]	Reviews digital tools for dietary monitoring, planning, and precision nutrition.	Provides field-level motivation and implementation context, but does not propose a concrete uncertainty-aware nutrient-estimation algorithm from food images.	Motivates the need for low-burden and repeated dietary assessment.
Image-based dietary assessment reviews	Chotwanvirat et al., 2024 [30]; Zheng et al., 2024 [31]; Dalakleidi et al., 2022 [32]; Shonkoff et al., 2023 [33]	Summarizes food-image recognition, segmentation, portion estimation, and nutrient calculation.	Highlights heterogeneous datasets, validation protocols, and error metrics, with limited attention to user-level personalized uncertainty propagation.	Defines the broader methodological landscape addressed by this study.
Food recognition and nutrient prediction	Tahir and Loo, 2021 [34]; Konstantakopoulos et al., 2024 [35]; Zhao et al., 2024 [36]; Wang et al., 2024 [37]	Improves visual recognition, segmentation, or direct nutrient prediction from food images.	Often emphasizes model accuracy or direct prediction, while calibrated uncertainty propagation and dietary-history personalization are less central.	Provides the visual-inference foundation on which downstream nutrient estimation can be built.
Depth- or camera-assisted intake estimation	Shao et al., 2023 [38]; Pfisterer et al., 2022 [39]; Van Wymelbeke-Delannoy et al., 2022 [40]; Whitton et al., 2024 [27]	Uses RGB-D, depth-refined segmentation, camera sensors, or technology-assisted protocols for portion or intake estimation.	Stronger portion information may require additional sensors, controlled meal settings, or structured institutional environments.	Clarifies why the present study focuses on nutrient-density and declared-reference errors when weighed intake is unavailable.
Foundation-model-based dietary assessment	Lo et al., 2024 [41]; O’Hara et al., 2025 [42]; Hoang et al., 2023 [43]	Uses multimodal or text-based AI systems to estimate food or nutrient information.	Outputs can depend on prompt design, model version, context, and implicit reasoning, making calibration and reproducible uncertainty evaluation difficult.	Highlights the value of explicit probability-based and reference-based uncertainty handling.
Personalization and uncertainty quantification	Saerens et al., 2002 [20]; Lipton et al., 2018 [21]; Alexandari et al., 2020 [22]; Angelopoulos and Bates, 2023 [17]; Vazquez and Facelli, 2022 [44]	Provides foundations for prior-shift correction and conformal uncertainty estimation.	These methods are usually not developed specifically for dietary-history-based nutrient-density estimation from food images.	Supplies the statistical basis for the prior-corrected and conformal components used here.
This study: CP-BayesNE Present study	—	Develops an uncertainty-aware personalized nutrient-density estimation framework from food images by combining calibrated food recognition, APS/RAPS (Adaptive Prediction Sets/Regularized Adaptive Prediction Sets) candidate sets, training-prior-corrected dietary-history personalization, posterior-weighted nutrient estimation, and separately calibrated nutrient-value intervals.	Does not claim direct true-intake accuracy because weighed portion references are unavailable; real-meal validation is limited to a single participant and declared nutrient references.	Integrates uncertainty representation and dietary-history personalization into a transparent downstream dietary-inference layer, and evaluates it using UEC-256, FoodLogAthl-218, and a 92-meal personal dataset.

**Table 2:** Dataset characteristics and analytical roles.

Dataset	Scale	Portion GT	Primary Role
UEC FOOD 256	256 classes; validation n=4592	No	Classifier development, calibration, APS/RAPS evaluation
FoodLogAthl-218	218 raw categories; 100 mapped; 51 users	No	Population-level personalization validation using nutrient-density targets
Personal Meal Dataset	92 meals; 402 item records; 1 individual	No independent weighed GT	Real multi-item pipeline evaluation using dish-/product-level declared nutrient references

**Table 3:** Primary experimental conditions.

Condition	Candidate Set	Train-Prior Correction	Personal Prior	Additional Refinement
A: no prior	APS	Yes	No	None
B: with prior	APS	Yes	Yes	None
C: adaptive	APS	Yes	Yes	Nutrient-specific fallback; nutrient interval calibration
RAPS only	RAPS	N/A	N/A	Category-level coverage and set-size analysis
B-RAPS	RAPS	Yes	Yes	Secondary downstream sensitivity analysis for condition B

Note: N/A = not applicable.

**Table 4:** Food-recognition and probability-calibration performance on UEC-256.

Metric	Value
Top-1 accuracy	78.01%
Top-5 accuracy	93.67%
ECE before calibration	0.11238
ECE after calibration	0.04345
Relative ECE reduction	61.34%
Optimal temperature	0.905738

**Table 5:** Nutrient-density MAE across 51 users. Units are kcal/100 g for energy and g/100 g for macronutrients.

Condition	Energy (kcal/100 g)	Protein (g/100 g)	Fat (g/100 g)	Carbohydrate (g/100 g)
A: no prior	37.049	2.576	3.167	6.810
B: prior-corrected personalization	35.323	2.454	3.041	6.364
C: adaptive refinement	33.396	2.329	2.915	5.726

**Table 6:** Nutrient-density MAE under APS-constructed versus RAPS-constructed candidate sets (both under condition B, prior-corrected personalization). Units are kcal/100 g for energy and g/100 g for macronutrients. APS values reproduce Table 5.

Candidate Set	Energy (kcal/100 g)	Protein (g/100 g)	Fat (g/100 g)	Carbohydrate (g/100 g)
APS-B	35.323	2.454	3.041	6.364
RAPS-B	34.495	2.354	2.824	5.875
Relative change	−2.3%	−4.1%	−7.1%	−7.7%

**Table 7:** User-level paired statistics for prior-corrected personalization (condition B vs. condition A). These statistics predate the adaptive condition C. ΔMAE is defined as (condition B − condition A); a negative value indicates improvement. Wilcoxon signed-rank tests were two-sided; Holm-adjusted *p* accounts for simultaneous testing across the four nutrients.

Nutrient	Improved Users	Worsened Users	Median ΔMAE [IQR]	Wilcoxon *p*	Holm-Adjusted *p*
Energy	39	12	−1.379 [−2.604, −0.118]	$3\times 10^{-6}$	$6\times 10^{-6}$
Protein	43	8	−0.100 [−0.162, −0.040]	$1\times 10^{-6}$	$2\times 10^{-6}$
Fat	37	14	−0.087 [−0.238, 0.002]	0.000257	0.000257
Carbohydrate	45	6	−0.386 [−0.654, −0.175]	<$10^{-6}$	$3\times 10^{-7}$

**Table 8:** Personal 92-meal evaluation. Values are meal-level MAE under the available dish-/product-level declared nutrient references.

Condition	Energy (kcal)	Protein (g)	Fat (g)	Carbohydrate (g)
No prior + personal portion	323.10	14.27	14.64	34.06
Personal prior + adaptive refinement	313.44	14.14	15.12	27.03
Relative change	−3.0%	−0.9%	+3.3%	−20.6%

**Table 9:** Category-level conformal coverage under cross-domain and same-domain calibration (α=0.10), evaluated on the two independent real-photo domains. The UEC-256 row reproduces the same-domain reference from Table 4 for comparison.

Domain	Cross-Domain Coverage	Same-Domain Coverage	Avg. Candidate-Set Size
UEC-256 (reference)	—	99.35%	80.78
FoodLogAthl-218 (51 users)	90.84%	92.73%	106.45–117.55
Personal (92 meals)	92.75%	93.81%	100.68–103.11

## Data Availability

The UEC FOOD 256 and FoodLogAthl-218 datasets are available from their original providers under their respective access conditions. The FoodLogAthl-218-to-UEC-256 category mapping table, the UEC-256 nutrient-reference mapping table, and the analysis code generated in this study are available from the corresponding author upon reasonable request, subject to dataset licensing and institutional restrictions. The personal meal images are not publicly shared because they are self-collected dietary records from one author. Original images from third-party datasets are not redistributed in this article or by the authors.

## Abbreviations

The following abbreviations are used in this manuscript:

APS	Adaptive Prediction Sets
CP	Conformal Prediction
ECE	Expected Calibration Error
FA-218	FoodLogAthl-218
MAE	Mean Absolute Error
RAPS	Regularized Adaptive Prediction Sets
UEC-256	UEC FOOD 256
